# Virulence and in vitro antifungal susceptibility of *Candida albicans* and *Candida catenulata* from laying hens

**DOI:** 10.1007/s10123-020-00141-1

**Published:** 2020-08-09

**Authors:** Wafa Rhimi, Chioma Inyang Aneke, Giada Annoscia, Antonio Camarda, Adriana Mosca, Cinzia Cantacessi, Domenico Otranto, Claudia Cafarchia

**Affiliations:** 1 Dipartimento di Medicina Veterinaria, Università degli Studi di Bari “Aldo Moro”, 70010, Valenzano, Bari, Italy; 2 Department of Veterinary Pathology and Microbiology, University of Nigeria, Nsukka, 410001 Nigeria; 3 Dipartimento Interdisciplinare di Medicina, Università degli Studi di Bari “Aldo Moro”, Bari, Italy; 4 Department of Veterinary Medicine, University of Cambridge, Cambridge, UK

**Keywords:** *Candida albicans*, *Candida catenulata*, Laying hens, Virulence factors, Antifungal susceptibility

## Abstract

In spite of evidence that domestic and wild birds may act as carriers of human pathogenic fungi, data on the role of laying hens as reservoirs of drug resistant and virulent yeasts is lacking. Here, we assess several virulence factors (phospholipase and haemolysin activity) and the antifungal susceptibility profiles of 84 *Candida albicans* and 17 *Candida catenulata* strains isolated from cloacae (group A), faeces (group B) and eggs (group C) of laying hens. Of these strains, 95% *C. albicans* and 23% *C. catenulata* strains displayed phospholipase and haemolytic activities. For *C. albicans*, the highest values of phospholipase (Pz = 0.62) and haemolytic activities (Hz = 0.49) were recorded among the strains from group C whilst for *C. catenulata* (Pz = 0.54; Hz = 0.49) among those from group A. High minimum inhibitory concentration (MIC) values for azoles and amphotericin B (AmB) were recorded irrespective of their sources in all *C. albicans* strains. A total of 22 *C. albicans* strains were multidrug resistant, displaying resistance to fluconazole, itraconazole (ITZ), voriconazole (VOR) and posaconazole (POS). All *C. catenulata* strains from group C were resistant to ITZ, POS, micafungin and anidulafungin and susceptible to AmB. In this study, *C. albicans* and *C. catenulata* isolated from the cloacae, faeces and eggs of laying hens produced phospholipase and haemolysin and might be multidrug resistant. In the environment (faeces) or in eggs, *C. albicans* and *C. catenulata* strains might acquire pathogenic virulence traits and/or show multidrug resistance profiles. Based on these results, breeding and handling of laying hens and/or eggs may have implications for human and animal health.

## Introduction

*Candida* species are among the most common causes of infections, with overwhelming mortality rates in immunocompromised patients (Antinori et al. 2018; Tscherner et al. 2019). *Candida* spp. belong to the resident microbiota of the skin, mucosa and gastrointestinal tract of mammals including humans; in addition, domestic (Subramanya et al. 2017; Cafarchia et al. 2018) and wild birds (Brilhante et al. 2010; Brilhante et al. 2013) harbour and spread these yeasts in the environment via their faeces (Brilhante et al. 2010; Elhariri et al. 2015). Indeed, *Candida albicans* has been isolated from the gut, cloaca and droppings of domestic (e.g. laying hens, broiler chickens, pigeons) and wild (e.g. rhea, psittacinae, raptors, cockatiels) birds (Subramanya et al. 2017; Cafarchia et al. 2018; Brilhante et al. 2010). In addition, bird droppings act as a suitable substrate for the growth of *Candida* spp. due to intrinsically high concentrations of nitrogenous compounds (Elhariri et al. 2015). Yeasts such as *Candida*, *Cryptococcus*, *Geotrichum*, *Rhodotorula* and *Trichosporon* have also been isolated from environment, avian sources and eggs (Cafarchia et al. 2018). Interestingly, it has been demonstrated that laying hens might disseminate yeasts, mainly *C. albicans* and *Candida catenulata*, in the environment through eggs other than faeces (Cafarchia et al. 2018). At present, there is a growing concern that these yeasts species might acquire virulence determinants and antimicrobial resistance when moving between different niches (Tsiodras et al. 2008). Virulence factors such as secreted hydrolases (i.e. aspartyl proteases, phospholipases and haemolysins) play a pivotal role in the pathogenesis of *Candida* spp. infections (GunTang et al. 2017); the occurrence of such factors has been demonstrated in yeast strains, mainly *C. albicans*, isolated from the gut, cloaca and/or faeces of different avian species of synanthropic and pet birds or poultry (Cafarchia et al. 2008; Sidrim et al. 2010). Some of these yeasts also displayed a multidrug resistance profile (Brilhante et al. 2013; Sidrim et al. 2010; Lord et al. 2010; Brilhante et al. 2011; Brilhante et al. 2012), which has been associated, for fluconazole-resistance in particular, to the secretion of phospholipases (Ying and Chunyang 2012). In spite of the likely contribution of domestic and wild birds as carriers of human pathogenic fungi, no data is yet available on the role of laying hens as reservoirs of drug-resistant and virulent yeasts. Therefore, the present study aimed to assess (i) the production of hydrolytic enzymes such as phospholipase and haemolysin and (ii) the antifungal susceptibility profiles of C*. albicans* and *C. catenulata* isolated from the cloacae, faeces and eggs of laying hens.

## Materials and methods

### Fungal strains

A total of 101 strains of *C. albicans* (*n* = 84) and of *C. catenulata* (*n* = 17), previously isolated from the cloacae, faeces and eggs of laying hens (Cafarchia et al. 2018), were employed in this study. In particular, two or three *Candida* spp. strains per group in each positive shed were selected. *C. catenulata* was chosen since it has also been isolated from yolks and albumens of eggs (Cafarchia et al. 2018). The strains were assigned to three groups based on their origin. Group A included *C. albicans* (*n* = 67) and *C. catenulata* (*n* = 5) strains from cloacal swabs of laying hens; group B, *C. albicans* (*n* = 13) and *C. catenulata* (*n* = 4) strains from pools of faeces; and group C. *C. albicans* (*n* = 4) and *C. catenulata* (*n* = 8) strains from egg samples. The strains were isolated and identified based on colonial morphology, microscopic features, and by matrix-assisted laser desorption/ionisation time of flight mass spectrometry (MALDI-TOF MS), as previously reported (Cafarchia et al. 2018). Strains were stored at − 80 °C at the Department of Veterinary Medicine, University of Bari (Italy). Prior to testing, each strain was sub-cultured at least twice onto Sabouraud dextrose agar (SDA, Liofilchem, Italy) plates at 30 °C for 24–48 h to ensure strain purity and viability. All strains were molecularly confirmed by amplification and sequencing of the nuclear ribosomal ITS1-5.8S-ITS2 region.

In particular, genomic DNA was isolated from each strain using the DNeasy Blood & Tissue Kit (QIAGEN, Hilden, Germany), following manufacturer’s instructions. The nuclear ribosomal ITS1-5.8S-ITS2 region was amplified using ITS1 (5′-TCCGTAGGTGAACCTGCGG-3′) and ITS4 (5′-TCCTCCGCTTATTGATATGC-3′) primers. The PCR reaction consisted of 4 μl genomic DNA (∼ 100 ng) and 46 μl of PCR mix containing 2.5 mM MgCl_2_, 10 mM Tris-HCl (pH 8.3) and 50 mM KCl, 250 μM of each dNTP, 100 pmol of each primer and 1.25 U of AmpliTaq Gold (Applied Biosystems, Foster City, CA, USA), with an initial denaturation step of 10 min at 95 °C, followed by 35 cycles of denaturation for 1 min at 95 °C, annealing for 45 s at 55 °C, extension for 1 min 30 s at 72 °C and a final extension at 72 °C for 5 min. PCR products were examined on a 2% agarose gel stained with GelRed (VWR International PBI, Milano, Italy) and visualised on a Gel Logic 100 gel documentation system (Kodak, New York, USA). The amplicons were purified and sequenced in both directions using the Big Dye Terminator v.3.1 chemistry in a 3130 genetic analyser (Applied Biosystems, CA, USA). Sequences were aligned using the ClustalW program (Larkin et al. 2007) and compared with those available in GenBank by basic local alignment search tool (BLAST—http://blast.ncbi.nlm.nih.gov/Blast.cgi).

### Preparation of the yeast suspensions for the enzymatic activities

A loopful of the stock culture was streaked onto SDA and incubated at 37 °C for 24–48 h. Colonies were transferred into sterile saline solution and adjusted to an optical density of 1.3 using a turbidimeter (DEN-1McFarland Densitometer, Biosan) that was equivalent to 1.5 × 10^7^ CFU/ml as validated by quantitative plate count of colony forming unit (CFU) in SDA.

### Phospholipase activity

The production of phospholipase for *Candida* spp. was assessed using the egg-yolk plate method as previously described (Cafarchia et al. 2008; Price et al. 1982). A total of 10 μl yeast suspension was transferred onto egg-yolk plates and incubated at 32 °C for 5 days. The formation of a precipitation zone surrounding the colonies was considered as an indicator for enzyme production. The production of phospholipase (Pz) was expressed as a ratio of colony diameter by the total diameter of the colonies plus zone of precipitation (Price et al. 1982). Each strain was tested in duplicate, and the experiments were repeated over two consecutive days. The Pz values were reported as average of the values registered. The following ranges of activity according to the Pz index were established as follows: high, Pz ≤ 0.69; moderate, Pz = 0.70–0.89; weak, Pz = 0.90–0.99; none, Pz = 1 **(**Price et al. 1982**)**.

### Haemolytic activity

The haemolytic activity of *Candida* spp. was assessed by using SDA supplemented with 7% sheep blood and 3% glucose and adjusted to a pH of 5.6 ± 0.29. A total of 10 μl yeast suspension was inoculated in duplicate onto the media and incubated at 37 °C for 2 and 5 days, respectively. After incubation, a distinct translucent halo around the inoculation site indicated positive haemolytic activity (Elfeky and Gohar 2016). The ratio of the diameter of the colony (a) by that of the translucent zone (b) was used as value of the haemolytic index (Hz value) which represents the extent of haemolysin activity by the different *C. albicans* strains tested. Each strain was tested in duplicate, and the experiments were repeated over two consecutive days. The Hz values were reported as average of the values registered.

### Antifungal susceptibility test

The sensitivity to antifungal drugs of *C. albicans* and *C. catenulata* strains was evaluated using broth microdilution (BMD) testing in accordance with the guidelines available in the CLSI document M27-A3 (CLSI 2008). The following antifungal drugs were supplied by the manufacturers as pure standard compounds: amphotericin B (AmB) and itraconazole (ITC) (Sigma-Aldrich Milan, Italy), fluconazole (FLC) and voriconazole (VOR) (Pfizer Pharmaceuticals; Groton, Connecticut, USA), posaconazole (POS) (Schering-Plough Corporation, Kenilworth, NJ, USA), anidulafungin (ANI) (Sigma-Aldrich, Milan, Italy) and micafungin (MCF) (Sigma-Aldrich; Milan, Italy). FLC was dissolved in sterile water, whereas the remaining drugs were solubilised in dimethyl sulfoxide-DMSO (Sigma-Aldrich, Milan, Italy). The concentration of each antifungal drug ranged from 0.016 to 32 mg/L, except for FLC (i.e. from 0.06 to 128 mg/L). Visual reading of plates was performed after 48-h incubation at 35 °C. All strains were tested in duplicate. For azoles, the minimum inhibitory concentration (MIC) endpoint was defined as the lowest concentration that produced a significant decrease in turbidity (≥ 50%) compared with that of drug-free control. For AmB, the MIC endpoint was defined as the minimum concentration at which no visible growth was detected. Control strains (*Candida parapsilosis* ATCC 22019 and *Candida krusei* ATCC 6258 of the American Type Culture Collection, Manassas, VA, USA) were included to evaluate the accuracy of drug dilutions and reproducibility of results (CLSI 2008). Data were reported as MIC range, mean MIC value (MICm) and MIC to which 50% (MIC50) and 90% (MIC90) strains were inhibited. The following breakpoints or epidemiological cut-off values (ECV) were used to categorise these *Candida* strains as sensitive (S), dose-dependent sensitive (SDD) or resistant (R) (Pfaller 2012a; CLSI 2018): FLC (S MIC ≤ 2 μg/mL, SDD MIC = 4–32 μg/mL, R MIC ≥ 8 μg/mL); ITZ (S MIC = 0.125 μg/mL, SDD MIC = 0.25–0.5 μg/mL, R MIC ≥ 1 μg/mL); POS (ECV MIC ≤ 0.06 μg/mL, R MIC ≥ 0.06 μg/mL); VOR (S MIC ≤ 0.125 μg/mL, SDD MIC = 0.25–0.5 μg/mL, R MIC ≥ μg/mL); MCF and ANI (S MIC ≤ 0. 25 μg/mL, SDD MIC = 0.5 μg/mL, R MIC ≥ 1 μg/mL) and AmB (ECV S MIC ≤ 2 μg/mL, R MIC > 2 μg /mL).

### Statistical analyses

The Chi-square test (*χ*2) and the Student’s *t* test were used to compare the virulence data, resistance occurrence and mean MIC values of azoles, echinocandins, and AmB of *C. albicans* and *C. catenulata* from the three groups, respectively. A value of *P* < 0.05 was considered statistically significant.

## Results

The PCR assay produced species-specific bands of the expected size (550 bp) for both *C. albicans* and *C. catenulata* strains, and sequence analysis confirmed the initial identification, based on colonial morphology, microscopic features and MALDI identification as *C. albicans* (accession numbers: KY101860, MH061333, MN419374) for 84 yeast isolates and *C. catenulata* (accession number: KY103373) for 17 yeast isolates.

A total of 80 *C. albicans* (95%) and 4 *C. catenulata* (23.5 %) strains showed phospholipase and haemolytic activities that differed according to the sample source, with the highest values recorded for *C. albicans* (Pz = 0.62, Hz = 0.49) in strains from group C and for *C. catenulata* (Pz = 0.54; Hz = 0.49) in strains from group A (Table 1).

**Table 1:** Phospholipase activity and haemolysin activity of *Candida albicans* and *Candida catenulata* strains from cloaca swabs (group A), faeces (group B) and eggs (group C)

Group	*Candida albicans*				*Candida catenulata*			
	Phospholipase		Haemolysin		Phospholipase		Haemolysin	
	Number of isolates (%)	Mean Pz (sd)	Number of isolates (%)	Mean Hz (sd)	Number of isolates (%)	Mean Pz (sd)	Number of isolates (%)	Mean Hz (sd)
Group A	65/67(97.0)	0.65 (0.11)^a, b^	66/67 (98.5)	0.58 (0.12)	5/5 (100)	0.54 (0.03)^d^	2/5 (40)	0.49 (0.001)^e^
Group B	11/13 (84.6)	0.73 (0.03)^a^	11/13 (84.6)	0.52 (0.08)	2/4 (50)	0.72 (0.03)^d^	24 (50)	0.59 (0.002)^e^
Group C	3/4 (75.0)	0.62 (0.12)^b^	4/4 (100)	0.49 (0.02)	1/8 (12.5)	0.67 (0.04)^d^	0/8 (0)	0^e^
Total	80/84 (95. 2)	0.66 (0.05)	81/84 (96.4)	0.53 (0.04)	8/17 (47)	0.64 (0.09)	4/17 (23.5)	0.54 (0.07)

sd, standard deviation; the statistically significant differences were reported with the same superscript letters

High MIC values for azoles and AmB were recorded in all *C. albicans* strains from groups B and C (Table 2). *C. catenulata* showed high ITZ, POS ad VOR MIC values in strains from group B (faeces). ANI and MCF displayed the lowest MICs for both *C. albicans* and *C. catenulata* (Table 2). For *C. catenulata,* high MIC values for MCF (MIC _90_ = 8) and ANI (MIC _90_ = 8) were registered in strains from group C.

**Table 2:** Minimum inhibitory concentration (MIC, μg /mL) for the antifungal drugs tested of *Candida albicans* and *Candida catenulata* isolated from cloacal swabs (group A), faeces (group B) and eggs (group C) of laying hens

Groups	Antifungal agents	*Candida albicans*				*Candida catenulata*			
		Range	MIC_50_	MIC _90_	MIC m (dev.st)	Range	MIC_50_	MIC _90_	MIC m (dev.st)
Group A: cloacal swab	FLC	0.008-128	0.25	128	33.8 (55.4)	4–128	8	8	31.2 (54.1)^e^
	ITZ	0.06–32	0.25	32	7.6 (12.9)	0.25–32	0.5	1	6.9 (14.0)
	VOR	0.06–32	0.015	32	7.5 (13.1)	0.03–32	0.06	0.125	6.4 (14.2)
	POS	0.03–32	0.125	32	7.2 (12.7)	0.032–32	0.5	0.5	6.7 (14.1)
	MCF	0.03–1	0.03	0.125	0.1 (0.2)^a^	0.016–0.125	0.032	0.125	0.07 (0.05)
	ANI	0.03–0.5	0.06	0.5	0.1 (0.1)^c^	0.016–0.25	0.06	0.25	0.1 (0.1)^h^
	AmB	0.016–2	1	1	1.3 (0.5)	0.5–2	0.5	2	1.2 (0.75)
Group B: faeces	FLC	0.25–128	8	128	52.9 (61.8)	4–128	8	128	67 (70.4)^e^
	ITZ	0.25–32	1	32	10.3 (15.1)	0.25–32	0.5	32	16.1 (18.2)
	VOR	0.03–32	0.125	32	9.9 (15.3)	0.125–32	16	32	20.0 (15.2)^f^
	POS	0.06–32	0.5	32	7.6 (13.9)	0.5–32	16	32	20.1 (15.1)^g^
	MCF	0.03–0.125	0.06	0.125	0.06 (0.04)^b^	0.016–32	0.016	0.03	0.2 (0.4)
	ANI	0.03–0.125	0.125	0.25	0.1 (0.1)^d^	0.06–0.5	0.06	0.06	0.1 (0.2)^i^
	AmB	0.125–2	1	2	1.2 (0.6)	1–2	1	1	1.2 (0.5)^k^
Group C: eggs	FLC	4–128	128	128	67 (70.4)	8–32	32	32	19 (11.3)
	ITZ	0.25–16	16	16	8.3 (8.8)	1–8	2	8	3.8 (3.4)
	VOR	0.03–16	16	16	8.0 (9.2)	0.06–2	0.125	0.25	0.3 (0.7)^f^
	POS	0.125–16	16	16	8.1 (9.1)	1–4	2	4	2.6 (1.2)^g^
	MCF	0.06–2	2	2	1.0 (1.1)^ab^	2–16	2	8	4.7 (5.0)
	ANI	0.25–0.5	0.25	0.25	0.3 (0.1)^cd^	1–8	4	8	3.6 (2.1)^h,i^
	AmB	0.5–2	0.5	0.5	0.8 (0.8)	0.5–1	0.5	1	0.68 (0.3)^k^

FLC, fluconazole; ITZ, itraconazole; VOR, voriconazole; POS, posaconazole; MCF, micafungin; ANI, anidulafungin; AmB, amphotericin. The statistically significant differences were reported with the same superscript letter

A relatively large number of *C. albicans* strains from groups B and C were resistant to azoles (i.e. from 30.7% for VOR to 92.3 % for POS in group B and from 50 to 100% for all azoles in group C). MCF resistance was observed in *C albicans* strains from group A (6%) and group C (50%) (Table 3). The drugs with the highest number of resistant *C. albicans* strains was POS (i.e. 83.6% strains in group A, 100% strains in groups B and C) followed by FLZ and ITZ (Table 3). All *C. albicans* strains were susceptible to ANI and AmB. A total of 21 *C. albicans* strains (15 from group A, 4 from group B and 2 from group C) were multidrug resistant, displaying resistance to FLC, ITZ, VOR, and POS. All *C. catenulata* strains from the group C were resistant to FLZ, ITZ, POS, MCF and ANI but susceptible to AmB (Table 3). *C. catenulata* strains from group A (40%) and group B (25%) were resistant to AmB.

**Table 3:** *Candida albicans* and *Candida catenulata* strains isolated from cloacal swabs (group A), faeces (group B) and eggs (Group C) of laying hens that are resistant to at least one of the antifungal drugs tested

Antifungal agents	Group A: cloacal swab		Group B: pool of faeces		Group C: eggs	
	Number of isolates (%)		Number of isolates (%)		Number of isolates (%)	
	*C. albicans*	*C. catenulata*	*C. albicans*	*C. catenulata*	*C. albicans*	*C. catenulate*
FLC	24/67 (35.8%)^a^	4/5 (80%)	10/13(76.9%)^a^	3/4 (75%)	3/4 (75%)	8/8 (100%)
ITZ	25/67 (37.3%)	3/5 (60%)	7/13 (53.8)	2/4 (50%)	3/4 (75%)	8/8 (100%)
VOR	18/67 (26.8%)	1/5 (20%)	4/13 (30.7)	1/4 (25%)	2/4 (50%)	1/8 (12.5%)
POS	56/67 (83.6%)	4/5 (80%)	12/13 (92.3%)	4/4 (100%)	4/4 (100%)	8/8 (100%)
MCF	4/67 (6%)^b^	0/5 (0%)	0/13 (0%)	1/4 (25%)	2/4 (50%)^b^	8/8 (100%)
ANI	0/67 (0%)	0/5 (0%)	0/13 (0%)	0/4 (0%)	0/4 (0%)	8/8 (100%)
AmB	0/67 (0%)	2/5 (40%)	0/13 (0%)	1/4 (25%)	0/4 (0%)	0/8 (0%)

FLC, fluconazole; ITZ, itraconazole; VOR, voriconazole; POS, posaconazole; MCF, micafungin; ANI, anidulafungin; AmB, amphotericin. The statistically significant differences were reported with the same superscript letter

## Discussion

The results of this study demonstrate that *C. albicans* and *C. catenulata* isolated from cloacae, faeces and eggs of laying hens produce phospholipase and haemolysin and are resistant to azoles or AmB and echinocandin, thus highlighting the role of laying hens in harbouring and spreading pathogenic organisms in the environment. This finding is similar to that previously reported for strains from wild (Brilhante et al. 2013; Lord et al. 2010) or domesticated/pet birds (Subramanya et al. 2017; Ying and Chunyang 2012).

The production of phospholipase and proteinase has been shown to play a significant role in the pathogenesis and virulence of *Candida* species and related diseases (Dabiri et al. 2018). In addition, multidrug resistant yeasts often cause fatal infection in immunocompromised hosts (Mellinghoff et al. 2018). In this study, the virulence, azoles and echinocandins resistance profiles of yeasts varied according to the origin of *Candida* spp. In particular, for *C. albicans*, multidrug resistant strains were all isolated from the cloacae, faeces and eggs of laying hens, thus suggesting that the cloaca is the most likely source of multidrug resistance strains that are spread in the environment via the faeces or food. Resistance to azole derivatives is linked to changes in gene sequence or expression and appears to be linked to selective pressures induced by exposure to the azole products used in clinical practice or in agriculture (Brilhante et al. 2013; Brilhante et al. 2012; Pfaller 2012b). Since the animals from this study had no history of previous treatment with antifungal drugs, the drug-resistance detected in this study might be associated with the presence of these compounds in the feed or fruits administered to these animals, in accordance with previous reports (Subramanya et al. 2017; Brilhante et al. 2010; Brilhante et al. 2013). Long-term laboratory maintenance of microorganisms might influence the expression of some virulence factors; nevertheless, in this study, we observed that the virulence of *Candida* strains varied according to sample origin. In particular, the phospholipase and haemolytic activities of *C. albicans* were higher in strains isolated from eggs than those cloaca isolated ones. This finding might be linked to the extracellular enzymes secreted by *C. albicans* that might vary according to different host factors associated to the ecosystem within which the yeast resides (Gil-Bona et al. 2018). Thus, *C. albicans* might acquire virulence traits when in the environment or/and in eggs. For *C. catenulata*, the presence of drug resistance phenomena was most frequently observed among strains isolated from eggs. This finding might be due to a positive selection of the environment on the multidrug resistance of *C. catenulata* strains (Sidrim et al. 2010). However, *C. catenulata* strains from eggs are less able to produce phospholipase or protease, thus indicating a lower pathogenicity compared with the strains isolated from cloaca and suggesting a lower risk of human or animal infections posed from strains from the environment. Indeed, *C. catenulata* is a natural contaminant of dairy products but is nonetheless rarely associated with invasive or skin infection in human (Crozier and Coats 1977; Sârbu et al. 2013; Çakır et al. 2019; Ha et al. 2018; Radosavljevic et al. 1999).

In particular, even if > 10^6^ *C. catenulata* CFU/gr have been detected in Australian Camembert or other European cheese (Roostita 1996), the yeast was identified as the causative agent in onychomycosis or vulvovaginal infections and in only three cases of candidaemia (i.e. in one patient with gastric cancer, in one hospitalised premature infant and in one cirrhotic patient with endocarditis) (Crozier and Coats 1977; Sârbu et al. 2013; Çakır et al. 2019; Ha et al. 2018; Radosavljevic et al. 1999).

However, even if *C. catenulata* is less pathogenic than *C. albicans*, its high distribution and low susceptibility to ITZ, POS, MCF and ANI as well as AmB might require a specific therapeutic protocol. A case of *C. catenulata* invasive infection was reported in a cancer patient that was successfully treated with intravenous AmB, following failed FLZ treatment (Radosavljevic et al. 1999). Nosocomial infections caused by *Candida* spp. have become more common in recent years, and those caused by non-*albicans Candida* spp. are rising proportionally more than those by *C. albicans*, being responsible for 35–65% of all candidaemia in the general patient population (Sharma et al. 2017). Food or beverages may act as sources of *Candida* spp. infection that, following ingestion, might enter the bloodstream and cause fungaemia (Benedict et al. 2016). Although clinical cases are rare and occur in unusual clinical circumstances, the presence of yeasts in food may be of significance. In the environment (faeces) or in eggs, *C. albicans* strains are more pathogenic and are characterised by protease and haemolysin production and azole resistance, whereas *C. catenulata* is characterised by a multidrug resistance profile. Interestingly, the high MIC values for AmB detected among *C. albicans* from faeces or eggs and in *C. catenulata* from cloaca swabs are in contrast with those previously reported for yeasts isolated from *Agapornis* birds and cockatiels that are bred under different conditions (Brilhante et al. 2010; Reis et al. 2018).

However, despite the high MIC values registered for AmB, resistance phenomena were detected only in *C. catenulata* from groups A and B, most likely due to the high value of ECV used (i.e. MIC > 2) or to the small number of samples tested. Since AmB and echinocandins are the first line of treatment for *Candida* fungaemia (Keane et al. 2018; Demir et al. 2019), resistance to these drugs should be not underestimated and needs to be further investigated using molecular approaches. The co-habitation of humans and laying hens and/or eggs may expose humans to multidrug-resistant and virulent yeasts. The breeding and handling of these animals may have consequently substantial implications for human and animal health. Therefore, high hygiene procedures in laying hens farm as well as the use of personal protective equipment might be important to prevent human (keepers/or visiting) and bird exposure to infectious propagules contaminating the environment and the eggs.

## References

[CR1] Antinori S, Corbellino M, Parravicini C (2018). Challenges in the diagnosis of invasive fungal infections in immunocompromised hosts. Curr Fungal Infect Rep.

[CR2] Benedict K, Chiller TM, Mody RK (2016). Invasive fungal infections acquired from contaminated food or nutritional supplements: a review of the literature. Food borne Pathog Dis.

[CR3] Brilhante RSN, Castelo-Branco DSCM, Soares GDP, Astete-Medrano DJ, Monteiro AJ, Cordeiro RA, Rocha MFG (2010). Characterization of the gastrointestinal yeast microbiota of cockatiels (Nymphicus hollandicus): a potential hazard to human health. J Med Microbiol.

[CR4] Brilhante RS, Paiva MA, Sampaio CM, Teixeira CE, Castelo-Branco DS, Leite JJ, Sidrim JJ (2011). Yeasts from Macrobrachium amazonicum: a focus on antifungal susceptibility and virulence factors of *Candida* spp. FEMS Microbiol Ecol.

[CR5] Brilhante RS, Castelo Branco DS, Duarte GP, Paiva MA, Teixeira CE, Zeferino JP, Rocha M (2012). F. Yeast microbiota of raptors: a possible tool for environmental monitoring. Environ. Microbiol. Rep..

[CR6] Brilhante RSN, de Alencar LP, de Aguiar CR, Castelo DDSCM, Teixeira CEC, de Brito MR, de Oliveira MF (2013). Detection of *Candida* species resistant to azoles in the microbiota of rheas (Rhea americana): possible implications for human and animal health. J Med Microbiol.

[CR7] Cafarchia C, Romito D, Coccioli C, Camarda A, Otranto D (2008). Phospholipase activity of yeasts from wild birds and possible implications for human disease. Med Mycol.

[CR8] Cafarchia C, Iatta R, Danesi P, Camarda A, Capelli G, Otranto D (2018). Yeasts isolated from cloacal swabs, feces, and eggs of laying hens. Med Mycol.

[CR9] Çakır SÇ, Çelebi S, Özkan H, Köksal N, Dorum BA, Yeşil E, Hacımustafaoğlu M (2019). Results of the use of micafungin in newborns. Mikrobiyol Bul.

[CR10] CLSI DOCUMENT M27-A3. Clinical and Laboratory Standards Institute. Reference method for broth dilution antifungal susceptibility testing of yeasts; Approved Standard–third edition. 2008.

[CR11] CLSI DOCUMENT M59: Epidemiological cut-off values for antifungal susceptibility testing. Clinical Laboratory Standards Institute, 2edition, 2018.

[CR12] Crozier WJ, Coats H (1977). A case of onychomycosis due to Candida ravautii. Aust J Derm.

[CR13] Dabiri S, Shams-Ghahfarokhi M, Razzaghi-Abyaneh M (2018). Comparative analysis of proteinase, phospholipase, hydrophobicity and biofilm forming ability in *Candida* species isolated from clinical specimens. J Mycol Med.

[CR14] Demir KK, Butler-Laporte G, Lee TC, Cheng MP (2019) Comparative effectiveness of amphotericin B, azoles, and echinocandins in the treatment of candidemia and invasive candidiasis: A Systematic Review and Network Meta-Analysis. Open Forum Infect Dis (6):S716–S716 10.1111/myc.13290 33894072

[CR15] Elfeky DS, Gohar NM (2016). Evaluation of virulence factors of *Candida* species isolated from superficial versus systemic candidiasis. Egypt J Med Microbiol.

[CR16] Elhariri M, Hamza D, Elhelw R, Refai M (2015). Love birds and cockatiels risk reservoir of *Cryptococcus neoformans*, a potential hazard to human health. J Vet Sci Med Diagn.

[CR17] Gil-Bona A, Amador-García A, Gil C, Monteoliva L (2018). The external face of *Candida* albicans: a proteomic view of the cell surface and the extracellular environment. J Proteome.

[CR18] GunTang W, Kamonvoradej N, Chomchat C, Suriyakan S, Sanit S, Wongwigkarn J, Lamlertthon S (2017). Prevalence and virulence factors of *Candida* spp. associated with blow flies. Asian Pac. J. Trop. Biomed..

[CR19] Ha MV, Choy MS, McCoy D, Fernandez N, Suh JS (2018). Candida catenulata candidaemia and possible endocarditis in a cirrhotic patient successfully de-escalated to oral fluconazole. J Clin Pharm Ther.

[CR20] Keane S, Geoghegan P, Povoa P, Nseir S, Rodriguez A, Martin-Loeches I (2018). Systematic review on the first line treatment of amphotericin B in critically ill adults with candidemia or invasive candidiasis. Expert Rev Anti-Infect Ther.

[CR21] Larkin MA, Blackshields G, Brown NP, Chenna R, McGettigan PA, McWilliam H, Thompson JD (2007). Clustal W and Clustal X version 2.0. Bioinformatics..

[CR22] Lord AT, Mohandas K, Somanath S, Ambu S (2010). Multidrug resistant yeasts in synanthropic wild birds. Ann Clin Microbiol Antimicrob.

[CR23] Mellinghoff SC, Cornely OA, Jung N (2018). Essentials in *Candida* bloodstream infection. Infection.

[CR24] Pfaller MA (2012). Diekema DJ 2012. Progress in antifungal susceptibility testing of *Candida* spp. by use of Clinical and Laboratory Standards Institute broth microdilution methods, 2010 to 2012. J Clin Microbiol.

[CR25] Pfaller MA (2012). Antifungal drug resistance: mechanisms, epidemiology, and consequences for treatment. Am J Med.

[CR26] Price MF, Wilkinson ID, Gentry LO (1982). Plate method for detection of phospholipase activity in Candida albicans. Med Mycol.

[CR27] Radosavljevic M, Koenig H, Letscher-Bru V, Waller J, Maloisel F, Lioure B, Herbrecht R (1999). *Candida catenulata* fungemia in a cancer patient. J Clin Microbiol.

[CR28] Reis EJ, Buscariolo F, Siqueira JP, Castilho EM, Almeida MT (2018). Agapornis sp. pet birds: Source of dissemination of azole-resistant yeasts. Med Mycol.

[CR29] Roostita R, Fleet GH (1996). The occurrence and growth of yeasts in Camembert and Blue-veined cheeses. Int J Food Microbiol.

[CR30] Sârbu I, Pelinescu D, Stoica I, Măruţescu L, Vassu T (2013). Phenotypic profiles of virulence in different Candida species isolated from vulvovaginal infections. Roum Arch Microbiol Immunol.

[CR31] Sharma Y, Chumber SK, Kaur M (2017). Studying the prevalence, species distribution, and detection of *in vitro* production of phospholipase from *Candida* isolated from cases of invasive candidiasis. J Global Infect Dis.

[CR32] Sidrim JJC, de Souza Collares DCB, Brilhante RSN, Soares GDP, Cordeiro RA, Monteiro AJ, Rocha MFG (2010). *Candida* species isolated from the gastrointestinal tract of cockatiels (*Nymphicus hollandicus*): in vitro antifungal susceptibility profile and phospholipase activity. Vet Microbiol.

[CR33] Subramanya SH, Baral BP, Sharan NK, Nayak N, Metok Y, Sathian B, Gokhale S (2017). Antifungal susceptibility and phenotypic virulence markers of *Candida* species isolated from Nepal. BMC Res Notes.

[CR34] Tscherner M, Giessen TW, Markey L, Kumamoto CA, Silver PA (2019). A synthetic system that senses *Candida albicans* and inhibits virulence factors. ACS Synth Biol.

[CR35] Tsiodras S, Kelesidis T, Kelesidis I, Bauchinger U, Falagas ME (2008). Human infections associated with wild birds. J Inf Secur.

[CR36] Ying S, Chunyang L (2012). Correlation between phospholipase of *Candida albicans* and resistance to fluconazole. Mycoses..

